# A mixed methods approach to exploring the moderating factors of implementation fidelity of the integrated chronic disease management model in South Africa

**DOI:** 10.1186/s12913-020-05455-4

**Published:** 2020-07-06

**Authors:** Limakatso Lebina, Tolu Oni, Olufunke A. Alaba, Mary Kawonga

**Affiliations:** 1 Perinatal HIV Research Unit (PHRU), SA MRC Soweto Matlosana Collaborating Centre for HIV/AIDS and TB, Faculty of Health Sciences, University of the Witwatersrand, Johannesburg, South Africa; 2 School of Public Health and Family Medicine, University of Cape Town, Cape Town, South Africa; 3 MRC Epidemiology Unit, University of Cambridge, Cambridge, UK; 4 Health Economics Unit, School of Public Health and Family Medicine, University of Cape Town, Cape Town, South Africa; 5 Department of Community Health, School of Public Health, Faculty of Health Sciences, University of the Witwatersrand, Johannesburg, South Africa

**Keywords:** Chronic care model, Ideal clinic, Primary healthcare, Contextual factors

## Abstract

**Background:**

Chronic care models like the Integrated Chronic Disease Management (ICDM) model strive to improve the efficiency and quality of care for patients with chronic diseases. However, there is a dearth of studies assessing the moderating factors of fidelity during the implementation of the ICDM model. The aim of this study is to assess moderating factors of implementation fidelity of the ICDM model.

**Methods:**

This was a cross-sectional mixed method study conducted in two health districts in South Africa. The process evaluation and implementation fidelity frameworks were used to guide the assessment of moderating factors influencing implementation fidelity of the ICDM model. We interviewed 30 purposively selected healthcare workers from four facilities (15 from each of the two facilities with lower and higher levels of implementation fidelity of the ICDM model). Data on facility characteristics were collected by observation and interviews. Linear regression and descriptive statistics were used to analyse quantitative data while qualitative data were analysed thematically.

**Results:**

The median age of participants was 36.5 (IQR: 30.8–45.5) years, and they had been in their roles for a median of 4.0 (IQR: 1.0–7.3) years. The moderating factors of implementation fidelity of the ICDM model were the existence of facilitation strategies (training and clinical mentorship); intervention complexity (healthcare worker, time and space integration); and participant responsiveness (observing operational efficiencies, compliance of patients and staff attitudes). One feature of the ICDM model that seemingly compromised fidelity was the inclusion of tuberculosis patients in the same stream (waiting areas, consultation rooms) as other patients with non-communicable diseases and those with HIV/AIDS with no clear infection control guidelines. Participants also suggested that poor adherence to any one component of the ICDM model affected the implementation of the other components. Contextual factors that affected fidelity included supply chain management, infrastructure, adequate staff, and balanced patient caseloads.

**Conclusion:**

There are multiple (context, participant responsiveness, intervention complexity and facilitation strategies) interrelated moderating factors influencing implementation fidelity of the ICDM model. Augmenting facilitation strategies (training and clinical mentorship) could further improve the degree of fidelity during the implementation of the ICDM model.

## Contributions to the literature

- Chronic diseases are a major cause of morbidity and mortality, yet, there is limited data on the implementation of chronic care management models in low and middle-income countries. This study provides timely information on the evaluation of moderating factors that affect fidelity (adherence) to the guidelines of a chronic care model in a middle-income country.
- The results of this study also presents approaches on what factors to be addressed in primary healthcare clinics to enhance fidelity.
- Knowledge on the moderating factors that affect the implementation of the chronic care model would enhance sustainability, scale-up and scale out of the model.

## Background

The World Health Organization (WHO) describes a health intervention as any activity performed with or for an individual or groups of people with the aim of assessing, improving, promoting and maintaining good health [1]. The implementation of complex health interventions requires a high degree of exactness (fidelity) to the original design if the intervention is to be effective [2, 3]. An intervention’s failure to achieve expected results cannot be attributed to design error if a degree-of-fidelity evaluation has not be performed [3, 4]. In the scale-up and scaling-out of interventions, even if adaptations are made to enhance relevance, the critical components of an intervention should be implemented with a high degree of fidelity to the original design [5]. A description of the intervention’s non-adaptable key components in the guidelines could promote implementation fidelity as it would make it easy to modify the flexible components only [5, 6].

The degree of fidelity during the implementation of an intervention can be greatly influenced contextual factors [1, 7]. Contextual factors are the distinctive characteristics of a society, community, particular group or individuals that can influence how interventions are adopted and implemented [8]. The consolidated framework for the evaluation of contexts in the implementation of complex interventions separates context into outer context (socio-economic and political environment), and inner setting (organizational structural features, networks and culture), as well as the process of implementation and intervention, and the implementing team’s characteristics [8]. Systematic reviews, mainly of studies conducted in developed countries, found facilitators of implementation of chronic care models include communication, provider knowledge on the principles, strong committed leadership, funding, patient participation and different stakeholders’ interest in collaboration [9–11].

Carroll et al. (2007) describe four fidelity moderating factors (intervention complexity, facilitation strategies, quality of delivery and participant responsiveness) in their conceptual framework for implementation fidelity. The four factors are outlined below [6].

### Intervention complexity

Simple interventions that are well described with sufficient specific information are more likely to have a high level of implementation fidelity compared to complex ones [6].

### Facilitation strategies

Training, the provision of guidelines and monitoring increases the level of fidelity [6].

### Quality of delivery

Poor delivery of the activities or components of an intervention will have an impact on the overall level of fidelity of implementation [6].

### Participant responsiveness

The degree of fidelity in the implementation is affected by the acceptability of that intervention to the implementers and the recipients of the intervention [6].

The factors discussed above are not detached, but interrelated, with one moderator potentially predicting the other [6]. Hasson et al. reviewed and modified Carroll’s conceptual framework to include two additional constructs, recruitment and context [12]. In their study, contextual factors directly affecting fidelity include the positive experience of staff with similar programmes, financial resources, support for the patients’ relatives and external collaborations [12]. Challenges with the recruitment of participants into the programme (unwillingness to participate and not meeting inclusion criteria) were also recognized as another moderating factor for fidelity [12]. There is a dearth of studies that assess moderating factors influencing implementation fidelity of chronic disease management models in low- and middle-income countries.

In South Africa, a middle-income country, the Department of Health implemented the integrated chronic disease management model (ICDM model) in 2011 [13, 14]. This followed the principles of the of Chronic Care Model (CCM) and Innovative Care for Chronic Conditions (ICCC) frameworks, which aim to enhance efficiency of health services and health outcomes for patients with chronic disease at primary healthcare (PHC) level [13, 14]. The ICDM model’s four major interrelated components are clinical supportive management (clinical mentorship), facility re-organization (administrative and patient flow for efficiency), assisted self-support (adherence support) and strengthening support systems [13]. The objectives of the ICDM model are to improve waiting times, cleanliness, the attitude of staff, the availability of medicine and equipment, and patient safety and quality of care [13]. The ICDM model incorporates both communicable diseases (HIV/AIDS and tuberculosis (TB)) and non-communicable diseases (diabetes, hypertension, asthma, mental health, chronic obstructive pulmonary disease [COPD], epilepsy) [13]. The implementation of the ICDM model delivered results such as improvements in patients’ records, compliance with clinical guidelines and better health outcomes [15, 16]. However, the implementation processes and outcomes (acceptability, adoption and sustainability) varied between health districts and health facilities [15–18].

We evaluated the implementation fidelity of the ICDM model in 16 facilities across two health districts, which were the pilot sites for ICDM implementation before scale-up in South Africa [19]. We found that the degree (level) of fidelity varied by district, and facility – two facilities had low (< 70%), six had medium (70–79%) and eight had high (80–89%) fidelity scores [19]. The objectives of this study were to assess the moderating factors affecting implementation fidelity of the ICDM model in those two districts, and the impact of facilities’ characteristics on fidelity. Specifically, this study describes the moderating factors and their perceived influence on implementation fidelity, from the perspective of the healthcare workers and administrators responsible for implementing the ICDM at PHC facilities in South Africa.

## Methods

### Study setting

This study was conducted in the two health districts, the Dr. Kenneth Kaunda (DKK) district in the North West province and the West Rand (WR) district in the Gauteng province. These two health districts were the pilot sites for the ICDM model implementation, and were the sites of our larger study on ICDM model implementation [13, 19]. The two districts differ with regard to disease burden, socio-economic status and population size, as summarized in Table 1 [20–23]. The South African National Department of Health plans to introduce national health insurance (NHI) to increase access to health services and to revitalize primary health care services [24]. In addition to this, an ideal clinic realization and maintenance (ICRM) programme was initiated, with additional room in the budget to support PHC facilities with adequate infrastructure, staff, medicines and supplies, as well as regular evaluations on performance as part of the primary healthcare re-engineering [25]. It is within this context that the PHC clinics are implementing the ICDM model

**Table 1:** Demographic and health indicators for Dr. Kenneth Kaunda and West Rand Health Districts

Indicator	Dr. Kenneth Kaunda District	West Rand District
Population	716,272	810,613
Unemployment rate	25.4%	28.6%
Deprivation Index	1.92	1.76
Literacy rate	89.6%	97.6%
Informal Housing	21%	19.2%
Health Facilities	1 Regional Hospital; 3 District Hospitals; 9 Community Health Centres; 27 PHC Clinics; 6 satellite clinics and 2 mobile clinics	1 Regional Hospital; 2 District Hospitals; 4 Community Health Centres; 39 PHC Clinics
PHC Nurse workload (clients per nurse per day)	24.5	26.1
PHC Doctor workload (clients per doctor per day)	13.2	25.3
TB Incidence per 100,000	696	440
TB Successful Treatment	60.1%	80.6%
Hypertension Prevalence	39.1%	36.1%
Mental Health admission rate	2.05%	1.5%

.

### Description of the intervention (ICDM model)

The ICDM model targets both adults and children who have communicable or non-communicable chronic diseases [13]. The main implementers of this chronic care model at facility level are administrators, primary healthcare nurses and medical officers (generalist doctors), ICDM champions (nurse advocates for ICDM model activities), the district clinical specialist team (DCST), ward-based outreach teams (WBOTs) and community healthcare workers (CHCWs) [13]. The main activities of the ICDM model are overall health services re-organization, strengthening of support structures (supply chain management), clinical management support (DCST) and assisted self-management (WBOTs and CHCW) [13].

The ICDM model activities are organized into four major components, these being facility re-organization, clinical supportive management, assisted self-management, and strengthening of support systems [13]. Facility re-organization entails the management of patient flow to improve operational efficiency, reducing waiting time and patient satisfaction with the health services [13]. The second component of the ICDM model promotes quality care for patients with chronic diseases and support for the healthcare workers with appropriate training, guidelines and clinical mentoring by the DCST [13]. The WBOTs and the CHCWs assist the patients with self-management of their chronic diseases and provide adherence monitoring, screening for complications and point-of-care testing in the community [13]. The ICDM model’s fourth component is aligned with the ideal clinic initiative of enhancing supply chain management and collaborations with other stakeholders, such as school health teams [13].

### Study design

The study used a cross-sectional mixed method as part of a larger protocol that assessed the fidelity and costs of implementing the ICDM model in 16 PHC clinics (8 in the WR and 8 in the DKK health districts). The full study design has been described elsewhere [26], and the findings of the fidelity assessment have also been presented in another manuscript [19]. The results of that fidelity assessment were used to select the PHC clinics for inclusion in this study.

As part of that broader study, the level of implementation fidelity of the ICDM model was assessed in the 16 facilities, using an 89-item fidelity score designed to measure adherence to nationally-recommended ICDM model activities grouped within four ICDM model components [19]. Following the process evaluation framework, we scored at each facility the level of adherence (fidelity) to each recommended activity [19]. Fidelity scores for each of the four components (component score) and the overall fidelity score (sum of component scores) were compared across facilities and between the two health districts [19]. We applied the South African Ideal clinic rating system [not achieved (< 70%), silver (70–79%), gold (80–89%) and platinum (90–100%)] [25] to interpret the degree of fidelity per facility and per district. The assessment found that the WR district had a higher median fidelity score than the DKK district [19].

Based on the results of the fidelity assessment [19], four facilities were selected for healthcare workers interviews on their perceptions of moderating factors for fidelity – in each district, one clinic with the highest and one with the lowest fidelity score. In the WR district, the two selected facilities had fidelity scores of 86.1% (136/158) and 76.6% (121/158), while in the DKK district the selected facilities had scores of 84.8% (134/158) and 65.8%(104/158) (Table 2). The modified implementation fidelity conceptual framework [6, 12] was applied in the four facilities for identifying potential moderators that may have influenced fidelity of implementation of the ICDM model.

**Table 2:** The degree of implementation fidelity of the integrated chronic disease management model for the four clinics that were selected for interviews with healthcare workers

	*Overall*	*Higher Fidelity level Clinics*	*Lower Fidelity Level Clinics*	*P-values*
***Level of fidelity in the implementation of the ICDM model Median (IQR)***				
Facility Reorganization (max*: 37)	29 (28–30)	28 (27–29)	30 (29–30)	0.2207
Clinical Supportive Management (max*: 39)	31 (25–35)	35 (33–37)	25 (20–29)	0.1213
Assisted Self-Management (max*: 39)	33 (29–37)	37 (36–39)	29 (27–30)	0.1213
Strengthening of Support Systems (max*: 43)	35 (30–36)	36 (34–37)	30 (25–35)	0.4386
Overall Fidelity score (max*: 158)	128 (113–135)	135 (134–136)	113 (104–121)	0.1213

* Max = maximum possible fidelity score

**Table 3:** *Perceptions of healthcare workers on the ICDM model principles and recommended activities for patients with chronic diseases*

Variable	Agree	Disagree	Undecided
**Facility re-organization**			
1. Time Integration	27 (90)	3 (10.0)	–
2. Consulting room space integration	22 (73.3)	8 (26.7)	–
3. Booking system integration	28 (93.3)	2 (6.7)	–
4. Medical records integration	29 (96.7)	1 (3.3)	
5. Pre-pack medication	22 (73.3)	7 (23.4)	1 (3.3)
6. Designated *waiting areas*	22 (73.3)	7 (23.4)	1 (3.3)
7. Designated *vital signs stations*	20 (66.7)	9 (30.0)	1 (3.3)
8. Designated *consultation rooms*	25 (83.3)	5 (16.7)	–
9. Segregation of patients maintains order	25 (83.4)	4 (13.3)	1 (3.3)
10. Patients with communicable diseases should be in separate waiting areas	22 (73.3)	5 (16.7)	3 (10.0)
**Clinical Supportive Management**			
11. Healthcare worker integration	21 (70)	8 (26.7)	1 (3.3)
12. Nurses allocated for chronic diseases patients manage all eight conditions effectively	27 (90.0)	2 (6.7)	1 (3.3)
13. Care for patients with chronic diseases is enhanced when attended to by one nurse	23 (76.7)	7 (23.3)	–
14. Nurses have adequate training to be able to manage all the eight chronic diseases	16 (53.3)	7 (23.4)	7 (23.3)
**Assisted self-management**			
15. The ward-based outreach teams contribute to the management of patients with chronic diseases	27 (90.0)	2 (6.7)	1 (3.3)
16. The community healthcare workers team contributes to the management of patients with chronic diseases	27 (90.0)	–	3 (10.0)
**General principles**			
17. Management of patients with chronic diseases has improved since the introduction of the ICDM model	24 (80.0)	2 (6.7)	4 (13.3)
18. Patients with chronic disease like the ICDM model principles	26 (86.7)	2 (6.6)	2 (6.7)
19. Recommend that the ICDM model should be implemented in all clinics in South Africa	27 (90.0)	1 (3.3)	2 (6.7)
20. Recommend that the ICDM model should be implemented in other countries	27 (90.0)	–	3 (10.0)

**Table 4:** Characteristics of the 16 facilities that had implementation fidelity of the integrated chronic disease management model assessments

Variable	Mean (SD)
Budget customized by clinic	1 (1)
Distance from the district offices in km	40 (28)
Facility area under roof	657 (667)
Number of consulting rooms	6 (2)
Number of Professional Nurses	8 (4)
Number of Enrolled Nurses	2 (2)
Number of Medical Officers	2 (2)
Number of Pharmacy Assistants	1 (1)
Nurse-Patient Ratio	394 (205)
Medical Officer-Patient Ratio	2182 (1420)
Number of total patients per month	3241 (1193)
Number of total patients above 20 years per month	2352 (861)
Number of TB Cases Diagnosed in a month	5 (5)
Monthly Diabetic consultations	68 (35)
Monthly mental health consultations	26 (32)

**Table 5:** Univariate Linear regression assessing the impact of facility characteristics on the implementation fidelity of the ICDM model

	Univariate		
Variable	ß (SE)	95% CI	*p*-value
Budget customized by clinic	9.50 (5.1)	−1.33 – 20.33	0.810
Distance from the district offices	−0.76 (0.1)	−2.96 – 0.14	0.473
Facility area under roof	0.01 (0.0)	−0.00 – 0.02	0.140
Number of consulting rooms	2.01 (1.2)	−0.63 – 4.65	0.125
Number of Professional Nurses	−0.17 (0.7)	−1.57 – 1.23	0.803
Number of Enrolled Nurses	1.88 (1.2)	−0.77 – 4.53	0.150
Number of Medical Officers	1.18 (1.9)	−2.82 – 5.17	0.539
Number of Pharmacy Assistants	2.00 (5.6)	−10.07 – 14.07	0.727
Nurse-Patient Ratio	0.01 (0.0)	−0.04 – 0.05	0.740
Medical Officer-Patient Ratio	−0.00 (0.0)	−0.005 – 0.004	0.768
Mean number of total patients per month	0.00 (0.0)	−0.00 – 0.01	0.740
Proportion of mean number of total patients above 20 years per month to total patients	−1.00 (0.4)	−1.80 – −0.21	0.017*
Proportion of mean monthly diabetic consultations to total patients	−2.16 (1.3)	−4.93 – 0.62	0.118
Proportion of mean monthly mental health consultations to total patients	−4.84 (2.6)	−10.39 – 0.71	0.082

* Statistically significant at the 0.05 level

Healthcare workers (nurses, administrators and ancillary staff) who provide services to patients with chronic diseases were purposively selected to participate in this study on moderating factors. They were considered eligible for inclusion if they had worked in the study facility for six or more months and were willing to provide written informed consent for participation. A total of 30 healthcare workers were interviewed from the four health facilities during August 2018 to March 2019, 15 from the two facilities that had highest implementation fidelity and 15 from facilities that had the lowest implementation fidelity.

The process evaluation framework was applied in collecting data on the characteristics of the 16 facilities as a guide to assess processes in implementation of complex interventions like the ICDM model, and impact of contextual factors [27].

### Data collection and measurement

The structured interview tool included standardized open-ended and closed fixed-response questions (see supplemental file). The first section of the interview guide collected data on the participants’ demographics such as age, current role in the facility and years in that role. In keeping with Carroll’s conceptual framework on implementation fidelity, as modified by Hassan et al. [6, 12], we also collected data on the potential moderators for implementation fidelity as outlined below.

*Intervention characteristics*: Participants were questioned on the features of the ICDM model (the four components and recommended activities) to determine which they felt were straightforward and which were vague, and their views on whether and how those features affected fidelity.

*Facilitation strategies*: The participants were questioned on what strategies at facility level they thought may have supported the implementation of the various activities of the ICDM model in their respective facilities. They were also asked to list some of the barriers experienced in implementing the ICDM model as recommended.

*Participant responsiveness*: The healthcare workers’ perceptions of the ICDM model principles (including, integration of all patients with chronic diseases, designated waiting areas, consultation rooms and vital signs stations for patients with chronic diseases) were evaluated using a Likert scale as follows: 1-strongly disagree, 2-disagree, 3-neither or undecided, 4- agree and 5- strongly agree. Although patients (users) were not included in this study, the measure of participants’ responsiveness with regard to users was assessed by measuring staff’s perceptions of patient responsiveness.

*Context*: In the qualitative component, participants in the study were asked to identify facility specific issues (context) that might hinder or support implementation fidelity of the ICDM model. In addition, quantitative data were collected from 16 facilities on facility characteristics such as budgeting style (consolidated for all clinics or customized by clinic), space (total area underroof), number of consulting rooms, numbers of staff members by category, workload (PHC headcount over a six-month period) and number of patients that received care for chronic conditions at the facility over the same time period. The choice of facility characteristics to include was based on the literature [9–11], and initial findings from the larger study. Data on the characteristics of the clinics was collected by direct observations, measurements and interviews with clinic and district level managers as recommended under the process evaluation framework [27].

*Quality of delivery* was not included in this assessment as there were no other programmes or studies that we could consult to benchmark the quality as recommended in the framework [6]. Recruitment was also not included as it was not applicable to this setting.

The data collection tools were piloted in a few facilities and revised for clarity prior to administration. The interviews were conducted by two trained research assistants according to the structured interview questionnaire (see supplemental file). Each participant was interviewed individually. Responses were written verbatim on paper-based answer sheets and the data were later captured into the REDCap electronic database [28]. The data quality management involved reviewing data for apparent discrepancies, incorrect data and missing variables prior to capturing and as part of data cleaning. The data were exported from REDCap into NVivo (version 12) and the Statistical Package for the Social Science (SPSS Inc., version 25.0) [29, 30].

### Data analysis

Descriptive statistics – medians with interquartile ranges (IQR) and proportions were used to analyse participants demographics and perceptions on the ICDM model principles. The Likert scale scores on participants perceptions of principles for agree and strongly agree were combined and those for strongly disagree and disagree were combined to simplify interpretation and the reporting of results. A deductive thematic analysis approach was used to identify and describe the potential moderating factors of implementation fidelity of the ICDM model. The thematic analysis followed the six steps recommended by Braun and Clarke [31] (familiarization, generating initial codes, searching, naming and reviewing themes and summarizing the findings). Coding was structured around predefined concepts based on the modified Carroll’s conceptual framework on implementation fidelity [6, 12], and literature [9]. One researcher analyzed all the data for codes, and combined code outputs into themes. The code outputs and themes were submitted for review and discussion with the other researchers. A few illustrative quotes were selected to represent the views of the participants on some of the ICDM model implementation fidelity moderating factors. Facilities-specific factors associated with fidelity to the ICDM model were evaluated using univariate regression where the parameter estimate, standard error, 95% confidence interval and *p*-values were determined. As there was only one facility-level factor associated with fidelity to the ICDM model guidelines on the univariate analysis, we were unable to perform multivariate analyses.

### Ethics approval

The study was approved by the Medical Human Research Ethics Committees of the University of the Witwatersrand (Ref: R14/49) and the University of Cape Town (Ref: 127/2018). Written informed consent was received from all participating healthcare workers.

## Results

### Participants’ demographics

The median age of the 30 healthcare workers that participated in the study was 36.5 (IQR: 30.8–45.5) years, and they had been in their current roles for a median of 4.0 (IQR: 1.0–7.3) years. The majority (80.0%; 24/30) of the participants were females. Half (50.0%, 15/30) were nurses; 26.7% (8/30) were administrative staff and 23.3% (7/30) were in the “others” category (management, counsellors, pharmacy assistants).

### Intervention complexity

*Facility reorganization*: Most (80.0%; 24/30) of the participants agreed that administrative integration (same-day, common booking system and medical records) of health services for patients with chronic disease and a separate stream of care with designated consulting rooms are appropriate and straightforward ICDM model principles to implement (Table 3). There was moderate support for using the same consulting room for all eight chronic conditions (73.3%; 22/30) and having a designated waiting area (73.3%; 22/30) and vital signs stations (66.7%; 20/30) for patients with chronic diseases.

The interviewed staff members found that consultation of patients with TB disease in the same stream (waiting areas, consultation rooms) as other patients with non-communicable diseases and those with HIV/AIDS were the features of the ICDM model that was vague and that compromised fidelity. The guidelines were not specific about when patients with TB should be incorporated into the chronic diseases stream. The participants’ opinion was that it should be detailed that patients with TB should be incorporated in the chronic disease management stream after they had initiated TB treatment and have been assessed to no longer be infectious.

*“TB patients are infectious and will infect the diabetes patients, a patient with TB must not mix with some other patients.”* (FI16–3; Nurse).

“*A TB patient comes with MDR (TB) then that patient should be separated from others because they might infect other patients.”* (FI007–8; Nurse).

The participants experienced the more complex elements of the ICDM model to be the highly administrative tasks and separating patients by different streams of care. The healthcare workers that were interviewed felt that this requires more staff. The current staff shortage was regarded as one of the limiting factors when implementing the recommended ICDM model with activities such as bookings, pre-packing of medication and designated stream of care for chronic patients with fidelity to the ICDM model guidelines.

*Clinical supportive management:* The recommendation that all patients with one of the eight chronic conditions have to be included in one stream and have to be attended to by one healthcare worker could result in low fidelity if the nurse does not have experience in managing all the conditions. Although 90.0% (27/30) of the participants agreed that one nurse would be able to effectively manage all eight conditions, there were still concerns that some of the conditions (TB, mental health) should not be integrated with all other conditions to be managed under the ICDM model. The reasons for the concerns included that mental health patient management is a tedious and specialized. Participants also highlighted that not all nurses are experienced in the management of all eight conditions included in the ICDM model, especially HIV/AIDS, TB, COPD and mental health. This makes it difficult to provide quality care for all patients.

### Fidelity facilitation strategies

*Training*: Participants viewed training of all staff (clinical and administrative) on the ICDM model principles as one of the factors that would foster adoption and the sustainability of high implementation fidelity to the model. Further to that, participants indicated that the nurses would need additional training for the management of patients with HIV/AIDS, mental health and COPD.

“*The management must make sure that nurses get proper training on ICDM to avoid making small mistakes. So, with training they going to improve and know exactly what to do and understand what they are doing.*” (FI4–2; Data Capturer).

“In this clinic we only have one specialist nurse and I think all nurses should (attend) adult primary healthcare (training)” (FI4–7; Nurse).

*Clinical mentoring*: A total of 73.3% (22/30) of the interviewees confirmed that the clinics had access to DCST, but only 46.7% agreed that the DCST provides clinical mentoring. DCST mentoring and support for the clinical management of patients with chronic diseases was stressed as an important facilitator in adhering to the ICDM model guidelines. Furthermore, clinical record audits by the DCST should be carried out as recommended with feedback on what should be improved. The participants also indicated that access to clinical advice from the DCST by phone would help with the clinical management of complicated cases.

*“We need support and mentoring especially for those with PHC, they (DCST) only come for audits and not supporting us*.” (FI4–1; Nurse).

### Participant responsiveness

*Compliance by patients*: The greatest challenge that some participants (46.7%; 14/30) felt affected the quality of delivery of ICDM model activities was patients’ poor attendance of scheduled appointments and poor adherence to prescribed medication for their conditions. For example, patients who have uncontrolled hypertension or diabetes or an unsuppressed HIV viral load cannot be included in the fast lane appointments or alternative medication pick-up lines. As a result, adherence by the clinics to the recommended ICDM model guideline activities for both assisted self-management (spaced and fast-lane appointments and adherence clubs), and facility reorganization (medication pre-packaging and pre-retrieval of medical records) was low. A total of 56.7% (17/30) of the participants viewed adherence clubs as beneficial to patients and 53.3% (16/30) viewed them as beneficial to clinic operational efficiency.

Participants mentioned that empowered patients who understand their conditions were a possible factor in patients’ willingness to be in different stream of care for chronic diseases and down-referral to adherence clubs. They establish profound relationships with the healthcare workers. Other participants indicated that if patient feedback and community engagement on the services provided is considered, that would also enhance fidelity to the ICDM model and patient satisfaction.

*“Patients defaults because they never adhere to their appointments and we had already pre-packed their medication but they never come”* (FI7–3; Nurse).

*“To teach people to adhere to their appointment date, because of retrieval of files. We retrieve 10 files and only two comes.”* (FI16–2; Data Capturer).

*Stigma and discrimination*: Another concern that was highlighted was that separate medical records, waiting areas, vital signs stations and queues would reveal the medical conditions of patients to other clinic attendees and this would stir up stigma and discrimination. The participants proposed that the ICDM model should provide guidance on how the segregation of patients into different various streams by reason for consultation should be achieved while preventing discrimination and stigmatization.

“*They feel like they are being isolated and they feel stigmatized and that other patients can see.*” (FI7–1; Nurse).

*“Stigma, if people see you in the queue and seeing you with a chronic patient file, so I think patients need their privacy, and separating is invading their privacy.”* (FI4–2; Data Capturer).

*Staff attitudes*: Participating staff members also indicated that there should be a structured change management process and willingness among employees to implement the ICDM model’s principles to improve adherence to its recommendations.

*Role clarification*: Although 90.0% (27/30) of the participants indicated that the CHCW and WBOTs contribute substantively to the management of patients with chronic diseases, they also indicated that overall performance in their roles is not easy to assess. They commented that the roles and key performance areas of the CHCW and WBOTs are not properly defined in the ICDM model.

*“With WBOT there isn’t clear what they are supposed to do in the clinic. Because there is still overflow of patients.”* (FI-6; Nurse).

### Context

*Adequate staff*: Providing sufficient staff members on a rotational basis would support a higher degree of fidelity in the implementation of the ICDM model. An example is that if the staff member allocated to the fast-lane (issuing of medication to booked and stable patients) is not on duty, that service would not be provided according to guidelines until there is sufficient staff.

“*We don’t have enough staff, even now we rely on nurses doing their community service.*” (FI11–6; Nurse).

*Supply chain management*: Lack of proper supply and management of batteries, booking books, printed materials on the chronic diseases, essential equipment and other consumables were also cited as factors that could reduce adherence to the ICDM model guidelines. The availability of technology to collect accurate data and to enhance communication between the clinic and the patients was thought to also potentially improve fidelity by enhancing precise bookings and adherence to clinic appointments.

*Balanced patient caseloads*: Staff members cited that high numbers of patients requesting services and inadequate staff and resources result in failure to adhere to ICDM guidelines. Secondly, participants felt that patients seeking services at facilities far from where they stay lead to low fidelity to the activity of CHCW and WBOT teams tracing defaulters.

*Infrastructure*: The healthcare workers indicated that due to the existing infrastructure (small waiting areas, few consulting rooms) of the clinics, it is difficult to implement four streams of care and have separate waiting areas, vital signs stations and consultation rooms designated only for patients with chronic diseases. Ample infrastructure, space and the design of the clinic were considered important pre-requisites to adherence to the prescribed ICDM model activities. Participants mentioned that a bigger filing space is also required to adequately implement the pre-retrieval of medical records.

*“Even now we combine acute patients with chronic because we do not have sufficient space.”* (FI11–5; Nurse).

“*The facility infrastructure should be revamped. Even now, we currently do not have water. Therefore, we need proper infrastructure, backup electricity, and water supply.”* (FI16–2; Administrator)

#### The impact of facility-level factors on ICDM model implementation fidelity

The characteristics of the participating health facilities are summarised in Table 4. The maximum score on the level of implementation fidelity at the sixteen facilities was 158, and the fidelity scores ranged from 101 to 136 (min, max), with a median score of 125 (IQR: 117–132). Univariate linear regression indicated that customizing the budget for each facility (ß = 9.50), and increasing in the number of consulting rooms (ß = 2.01), enrolled nurses (ß = 1.88), medical officers (ß = 1.18), and pharmacy assistants (ß = 2.0) are associated with an increase in the level of fidelity to the implementation of the ICDM model (Table 5). An increase in the proportion of patients over 20 years old and those consulting for diabetes and mental health correlate with a decrease in fidelity.

## Discussion

This study provides quantitative and qualitative information on the facilities and intervention’s interrelated moderating factors that affect implementation fidelity to the ICDM model. Time, space and healthcare worker integration and administrative tasks were some of the ICDM model features that need further clarification to enhance fidelity. There were concerns about nosocomial TB transmission if TB patients are included in one stream (staff and space integration) with all other patients with chronic diseases. Fidelity facilitation strategies (training and clinical mentorship) and participant responsiveness (empowered compliant patients and staff attitudes) were also highlighted as moderating factors that influence the fidelity. Adequate staff and infrastructure and observed efficiencies were stressed as some of the contextual moderating factors that foster fidelity to the guidelines. The qualitative results were consistent with some of the quantitative findings that adequate staff (pharmacy assistants, nurses and medical officers) and infrastructure (consulting rooms) are associated with a higher degree of fidelity in the implementation of the ICDM model.

There were concerns about nosocomial TB transmission to other patients with chronic diseases if TB patients are included in the same stream of care with all patients with chronic diseases. These concerns have also been raised at other TB and HIV single facility integration services, especially if the facilities are not designed to have adequate ventilation [32, 33]. WHO recommends both administrative (rapid identification, separation and treatment of TB patients) and environmental (ventilation systems, masks and ultraviolet germicidal irradiation lights) measures to minimize nosocomial TB transmission [34]. These strategies to prevent nosocomial TB transmission should be a critical pre-requisite in the implementation of the ICDM model, as the clinics diagnose a median of six new TB patients monthly and there is a high prevalence of drug-resistant (DR) TB and a decentralization of DR-TB services to PHC clinics in South Africa [35].

Training and clinical mentorship were mentioned as ICDM model implementation fidelity facilitators. These are similar to what was identified as facilitators in the implementation of chronic disease models in other studies such as appropriate data to support start-up and ongoing evaluations, effective clinical leadership and skills and training of healthcare workers [36–38]. Optimal clinical leadership has also been cited as a facilitator for adherence and sustainability of the ICDM model in another study [17]. The literature review illustrates that if there are no skilled and experienced staff to undertake the new proposed responsibilities, it would be difficult to adhere to guidelines [38].

Compliance to prescribed medication; patient adherence to appointments; and the attitudes and undefined roles of staff members were emphasized as moderating factors (participant responsiveness) of implementation fidelity. An intervention in healthcare should be acceptable to both patients and healthcare workers in order to be successfully implemented [38]. According to patients who had been interviewed in another study, they did not like the rigid appointment system under the ICDM model [39]. Acceptance and adoption of the chronic care models was also shown to be influenced by providing staff members with information in an appropriate manner to persuade them that the proposed intervention is beneficial [38]. The attitude of staff was also considered to be affecting the sustainability and acceptability of the ICDM model in other assessments [17, 39]. Clearly defined roles and communication within a multi-disciplinary team were considered crucial in the implementation of chronic care models [38]. Management is essential in supporting staff members throughout the change process [38]. In our study some of the concerns were that the ICDM model reinforces stigma and discrimination as it segregates patients by reason for consultation, and in other studies healthcare workers indicated that it reduces the stigma around HIV/AIDS patients when they are in one stream with patients with other chronic conditions [39].

Participants in this study emphasized that observing improvements in operational efficiency following the implementation of the ICDM model principles leads to high fidelity. The consistent use of recommended procedures and manuals on another chronic disease management model was also associated with high fidelity [37]. This, however, creates a vicious circle of cause and effect, as adherence to the ICDM model guidelines is dependent on other contextual factors.

Contextual factors that were identified as moderating factors for fidelity in the implementation of the ICDM model included adequate infrastructure, staff and supply chain management. Supply chain management, adequate staff and infrastructure were also identified as the most important factors to be addressed by the national and provincial departments of health in South Africa if the PHC facilities’ quality of services is to be improved [25]. Stock-outs of medication, malfunctioning or unavailable equipment (e.g. blood pressure machines) and consumables (pre-packaging bags) were also identifies as factors that affected efficiency under the ICDM model according to the providers and the patients [39].

The findings of systematic reviews of studies conducted in developed countries are comparable to the results of this study, in that financial resources (infrastructure and more personnel), leadership and acceptability of the model to staff and patients and training of the chronic disease management model are important to support implementation of the model [9–11]. The need for communication, and a culture that promotes quality improvements was not identified as important in this study unlike the findings from the systematic reviews. In addition, supply chain management identified as important in this middle-income setting did not emerge as a challenge in developed countries [9–11].

### Strengths and limitations

One of the strengths of this study was that we used a mixed method in our efforts to identify the moderating factors of implementation fidelity to the ICDM model. The interviews with the staff members who were implementing the ICDM model at the PHC facilities provided an end-users’ perspective on how adherence to the ICDM model guidelines can be enhanced. The study also included facilities with different levels of implementation fidelity, and as such minimized selection and exposure bias.

One of the limitations of this study was that the effect of patient perceptions of the ICDM model was not assessed, as this was beyond the scope of the study. PHC facilities’ implementation fidelity to the ICDM model could have been influenced by both the responsiveness of the patients and the implementers. In addition, the perceptions the healthcare workers shared could also have been influenced by social desirability bias. Their focus may have been to improve their working conditions and not necessarily patient-centred care. The study did not assess the potential impact of the differences in disease burden and socio-economic status between the two health districts on the ICDM model fidelity. Finally, the sample size of the health facilities included in the study was small from the perspective of conducting quantitative analysis. The small sample size limits the generalizability of the results. However the methods and findings could be applicable to other healthcare settings with similar characteristics.

## Conclusion

Our review of the ICDM model characteristics, fidelity facilitation strategies, participants’ responsiveness and the context has revealed a number of interrelated fidelity-moderating factors. These include time, space and healthcare worker integration, training, infrastructure, adequate staff and empowered compliant patients. The participants views suggest that addressing some of the moderating factors, such as supply chain management and leadership support, and enhancing facilitation strategies (training, clinical mentorship) could improve adherence to the ICDM model guidelines. As the PHC facilities observe the operational efficiency subsequent to following the ICDM model guidelines, they will be encouraged to increase the adoption and sustainability of the model. More research that includes a larger sample size could provide additional moderating factors that affect the implementation fidelity of the ICDM model.

## Data Availability

The dataset supporting the conclusions of this article is available in the Figshare repository, [10.6084/m9.figshare.11791176.v1].

## Abbreviations

CCM: Chronic Care Model
CHCWs: Community Healthcare workers
COPD: Chronic Obstructive Airway Disease
DCST: District Clinical Specialist Team
ICCC: Innovative Care for Chronic Conditions
ICDM: Integrated Chronic Disease Management
ICRM programme: Ideal clinic realization and maintenance programme
HIV: Human Immune Deficiency Syndrome
NHI: National Health Insurance
PHC: Primary Health Care
SA: South Africa
TB: Tuberculosis
WBOTs: Ward-based outreach teams
WHO: World Health Organization
